# Internal Hernia: An Uncommon and Often-Missed Differential Diagnosis of Abdominal Pain

**DOI:** 10.7759/cureus.75801

**Published:** 2024-12-16

**Authors:** Chaitanya Mishra, Khawaja O Omar

**Affiliations:** 1 Division of Pulmonary Critical Care Medicine, Charleston Area Medical Center, Charleston, USA

**Keywords:** gastric bypass surgery, gi radiology, high mortality, lactic acidosis, missed diagnosis, rare cause of acute abdominal pain, strangulated internal hernia

## Abstract

Abdominal pain is a common presenting symptom among patients visiting the hospital. A wide range of differential diagnoses are associated with this presentation, some of which are more uncommon than others, and require a higher degree of clinical suspicion and radiological excellence to diagnose. Although clinicians rely on physical assessment, examining a patient who is agitated and non-cooperative sometimes limits the physical exam findings, making these diagnoses even more challenging. We present a case of a middle-aged male patient who had acute abdominal pain in the setting of polysubstance abuse as confirmed by a drug screen. His initial imaging was unremarkable for a cause and the patient was intubated. After intubation and sedation, his physical examination was benign. He was extubated the next day and complained of worsening abdominal pain. His physical exam then was concerning for an acute abdomen and upon review of the initial imaging, a missed diagnosis of internal hernia was established. The patient subsequently underwent emergent exploratory laparotomy and a large section of the bowel was resected. This case highlights the importance of considering alternate diagnoses for common presentations and paying careful attention to classic radiology findings in these situations.

## Introduction

Internal hernias are the protrusion of abdominal viscera, most commonly small bowel loops, through a peritoneal or mesenteric opening into a compartment in the abdominal or pelvic cavity [1]. The openings may have normal (foramen of Winslow), paranormal (paraduodenal, ileocecal, supravesical fossa), and abnormal (transomental defect) anatomies [2]. The majority of internal hernias originate in congenital anomalies that have occurred during the internal rotation of organs. Other causes of internal hernias are atrophy of the omentum, dilation of the foramen of Winslow as a result of increased intra-abdominal pressure, inflammatory and ischemic processes of the cavity, and iatrogenic due to abdominal surgery [1]. Due to the intermittent nature of the symptoms, this condition is also challenging to diagnose. The classic CT findings include the swirled appearance of the mesenteric fat or vessels at the root of the mesentery, small bowel obstruction, clustered loops of small bowel, and mushroom shape of the herniated mesenteric root with crowding and stretching of the mesenteric vessels [3]. Due to its low incidence in the adult population, this cause is often forgotten by surgeons and radiologists as well, but it is becoming more common due to an increase in the quantity and types of abdominal surgeries [4]. This article discusses a case of initial missed diagnosis of an internal hernia which subsequently necessitated a major surgical resection and morbidity. The purpose of presenting this case report is to highlight this condition and improve its recognition. Although they have an overall incidence of 0.5-0.9% [5], internal hernias are associated with a high mortality rate of greater than 50% [6] and often go undiagnosed. They can either be congenital or acquired. With increasing Roux-en-Y gastric surgeries they are becoming more common [7]. Moreover, patients with acquired internal hernias tend to be older and have higher post-operative mortality compared to those with congenital ones [4].

## Case presentation

A 45-year-old male patient with a past medical history of substance abuse presented with dyspnea, abdominal pain radiating to the back, and diaphoresis. His symptoms had begun acutely an hour before the presentation. He complained of 10/10 abdominal pain. He was tachycardic with a heart rate of 124 beats per minute, tachypneic with a respiratory rate of 32 breaths/min, afebrile at 96.4°F, and a relatively stable blood pressure of 132/72 mmHg. He confirmed an active history of drug use and his urine drug screen was positive for amphetamine, opiate, and tetrahydrocannabinol. An abdominal exam per the ER physician revealed diffuse non-specific tenderness without definite peritoneal signs. With the acuity of pain, severe agitation, concern for arterial dissection requiring emergent imaging, and dyspnea with a high likelihood of impending respiratory failure, the ER physician decided to intubate the patient. The patient was also administered an initial volume challenge of 30 ml/kg crystalloid bolus. A CT angiogram of the chest and abdomen revealed no evidence of dissection or bowel obstruction on the initial review. The patient was admitted to the ICU. On clinical assessment, he had a soft abdomen. As the initial exam and radiological findings did not point toward any abdominal pathology, the surgery service was not consulted. His relevant lab data are given below (Table 1). 

**Table 1:** Laboratory data on presentation CRP, C-reactive protein

Category	Result	Normal range
White blood cell count (x10^3^/μL)	9.7	4–11
Creatinine (mg/dL)	0.9	0.7-1.3
Lipase (U/L)	15	0-160
Troponin (ng/ml)	0.003	0-0.04
Lactic acid (mmol/L)	2.7	<2
CRP (mg/dL)	0.5	<0.9
Procalcitonin (ng/ml)	0.05	<0.05

**Figure 1:**
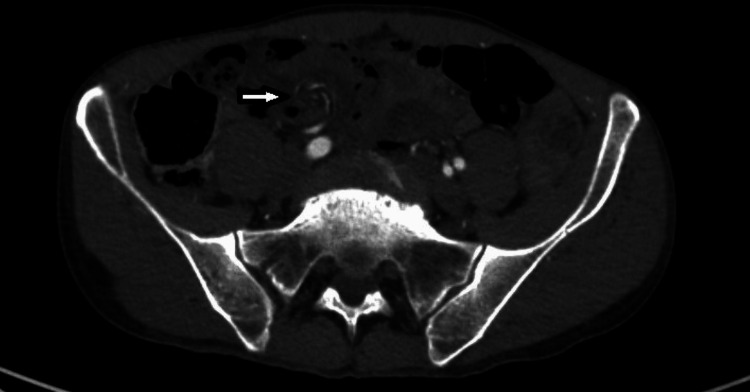
CTA abdomen pelvis demonstrating swirling of the mesentery (white arrow) CTA, CT angiography

**Figure 2:**
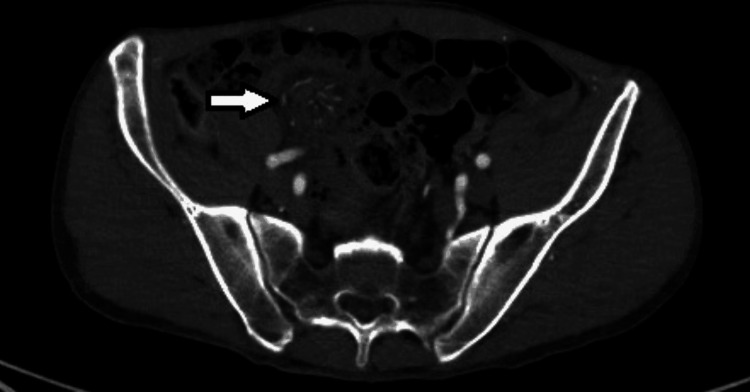
CTA abdomen pelvis demonstrating swirling of the mesentery (white arrow) CTA, CT angiography

The patient was clinically stable until the following day when he self-extubated and complained of recurrent abdominal pain. His lactic acid worsened to 4.7 mmol/L. An abdominal X-ray revealed a gaseous distention of several loops of small bowel concerning for obstruction. Urgent surgical evaluation was sought and the patient now had excruciating abdominal pain out of proportion to his clinical exam. After reviewing his imaging and post surgical assessment, a concern for a missed diagnosis of internal hernia on presentation was established (Figures 1, 2).

The patient was taken to the OR and found to have an internal hernia. He successfully underwent a resection of a large segment of necrotic small bowel. 

## Discussion

Internal hernias are a rare cause of intestinal obstruction. Due to their fluctuating symptoms, they present a diagnostic challenge both for the clinician as well as the radiologist. In cases where there are other confounding factors such as substance abuse and acute intoxication, establishing this diagnosis becomes even more daunting. Even without risk factors such as previous abdominal surgery, trauma, or peritoneal inflammation, the possibility of small bowel obstruction secondary to internal hernia should be considered [2]. 

The clinical presentation of this condition is typically non-specific. Vague abdominal discomfort, nausea, vomiting, abdominal distension, and recurrent obstruction are the common features. Worsening lactic acidosis is a sign of bowel ischemia and can be supportive of a diagnosis of internal hernia. The gold standard of imaging for this condition is a CT of the abdomen with contrast [8]. The sensitivity and specificity of a CT scan for this condition are about 80% and 85%, respectively [3]. Due to the urgency of this condition and the need for an accurate diagnosis, other modalities such as an MRI and an ultrasound of the abdomen are usually not pursued. When associated findings of bowel ischemia and hypoperfusion are present on imaging without a definite etiology, rarer diagnoses such as internal hernia should be considered, as it has a significant overall mortality rate exceeding 50% [6]. Complications of delayed diagnosis also include bowel ischemia, strangulation, and abdominal perforation. The treatment of this condition usually requires urgent surgical exploration with the extent of involvement of the bowel determining the best approach, i.e., laparoscopic vs open [4]. 

In this case, the patient presented with a confounding history of drug intoxication, and his initial workup with imaging and blood work were unrevealing for an acute pathology. As he was intubated and sedated, his clinical exam was limited. Due to this benign workup and confounding condition, a surgical evaluation was not sought initially. The following day, the patient had a recurrence of symptoms and an abdominal X-ray revealed a bowel obstruction which was not present on admission. A surgical consult was then requested. Upon reviewing the admission CT angiography, it became evident that the patient had a swirling mesentery on presentation which was suggestive of an internal hernia. This highlights the need for a multidisciplinary approach, right from the initial presentation, toward cases with complex presentations and fluctuating symptoms with likely better outcomes due to a discussion amongst various specialties including radiology, surgery, and emergency services. 

Anchoring bias is a significant limitation in medicine. In a recent study Ly et al. showed how once a diagnosis was attached to a patient on presentation, it delayed the findings of other conditions in the emergency room (ER) [9]. Our case also had a similar limitation where once the diagnosis of polysubstance abuse and intoxication was established in the ER, it made the diagnosis of internal hernia more challenging. 

## Conclusions

Many different clinical approaches and algorithms have been described for abdominal pain in literature which primarily include serial abdominal exams, imaging, and lab work. This case highlights a diagnosis that is uncommon and potentially easily missed. Although representing a small percentage of causes of abdominal pain, the incidence of internal hernia is increasing due to iatrogenic causes. By reporting this case, we seek to highlight the importance of timely diagnosis of this condition to prevent associated morbidity. It also brings to light the importance of re-evaluating the data when it does not correlate with the patient’s clinical picture and requesting appropriate multidisciplinary consult and review of complex cases in the ER early on during the presentation. We also wish to highlight the detrimental effect of anchoring bias in clinical medicine and the need to independently review facts and data as they become available. 
